# Climate change at a crossroads: Embedding environmental sustainability into the core of pharmacy education

**DOI:** 10.1177/17151635231152882

**Published:** 2023-02-15

**Authors:** Annalise Mathers, Shirley Fan, Zubin Austin

**Affiliations:** University of Toronto, Leslie L. Dan Faculty of Pharmacy, Toronto; University of Toronto, Leslie L. Dan Faculty of Pharmacy, Toronto; University of Waterloo, School of Pharmacy, Waterloo, Ontario; University of Toronto, Leslie L. Dan Faculty of Pharmacy, Toronto

In case it has miraculously escaped your attention, climate change is at a critical juncture. For decades, the alarm bells have been ringing: climate change poses an enormous threat—some would say, the greatest threat^1^—to global human health.^2^ Yet there has been very little substantive action to reconcile the perilous changes that are happening to our global ecosystem and establish a foundation for future sustainability.

While the link between the impact of climate change on services and systems related to human health is becoming more evident through increasingly frequent extreme weather events, contaminated water supplies, the exacerbation of respiratory and cardiovascular diseases and the spread of infectious diseases,^3,4^ the impact of our health care system as one of the largest contributors to climate change has only recently become more apparent.^4^ The research literature has evidenced the significant harm that our health care systems, and particularly pharmaceutical industries, contribute to our environment, magnifying the impacts of climate degradation.^2,4^ In Canada, our health care systems are responsible for 5% of all greenhouse gas emissions, and pharmaceuticals comprise 25% of these.^5^ In addition, the inadequate disposal of pharmaceutical waste has led to contamination and bioaccumulation in our water and food systems.^6-8^ These significant and harmful impacts of our health care systems on the environment and on climate change are only worsening.

Although the substantial adverse impacts of the pharmaceutical sector on climate are increasingly well known, a smaller body of research has examined the role of pharmacists in the climate crisis.^9-11^ As the health care providers who have the most direct contact with patients, pharmacists play a pivotal role as experts on how medications affect our bodies and, in theory, our planet.^11^ Recent studies hint at the lack of awareness and need for increased environmental training among pharmacists.^12-14^ Furthermore, while other studies show that pharmacy students are willing and able to engage in prioritizing sustainable practices, our current climate crisis needs much more than good intentions; it requires substantive knowledge and skill development in the training of our emerging pharmacy professionals.^15^

At present, there is no consensus or mandatory requirements among pharmacy schools in Canada, or elsewhere in the world, detailing the specific environmental sustainability competencies and skills a pharmacist should possess.^16^ Some schools and organizations have provided position statements and the need to teach “green” environmental sustainability principles.^17^ One example often quoted is the Association for Medical Education in Europe, which published a statement to “provide a global, collaborative, representative and inclusive vision for educating an interprofessional health care workforce that can deliver sustainable health care and promote planetary health.”^18^ Despite these declarations, true implementation of pharmacy environmental sustainability education is lacking.^10,17^ There is a strong need to move beyond the simple stance that acknowledges climate change is, indeed, a threat and move into an aligned, “collaborative call to action.”^19^ At present, the absence of unified education and training for pharmacists to combat the climate crisis represents a direct threat to the crisis itself.

So, what approaches do exist to equip emerging pharmacy professionals with the knowledge and skills paramount to leading an environmentally sustainable future? While much more work needs to be done, there are some promising practices to learn from. For current practising pharmacists, Roy^11^ details 8 practical actions by which pharmacists in Canada and globally can act as ambassadors for environmental sustainability, including using environmentally friendly inhalers and anesthetics, optimizing the use of medications, providing education on medication disposal and sustainable health care, advocating to national health care leadership and others.

For emerging pharmacy professionals, current initiatives focus on environmental sustainability being integrated into the curriculum,^20,21^ embedded into mandatory coursework^19,22,23^ via elective courses^24,25^ or as extracurricular activities.^26-28^ These approaches have not yet been validated or scaled across more than 1 university setting, nor do they close the gap of what pharmacists need to know, and do, to combat the climate crisis. See Table 1 for some examples of approaches to implementation of environmental sustainability into pharmacy curricula.

**Table 1:** Examples of approaches to implementation of environmental sustainability into pharmacy curricula around the world

**Institution**	**Description**
Approach 1: Integration of climate and health across the entire curriculum	
University of Huddersfield Department of Pharmacy (UK)^20^	• Embedded environmental sustainability principles into the pharmacy degree (MPharm) curriculum • Emphasis on reframing pharmacy topics through an explicit environmental lens; teaching on climate change is provided through extracurricular talks from experts in this field, offering related research opportunities for pharmacy students
Helsinki University Pharmacy Faculty (Finland)^21^	• Developed a new curriculum in 2016 integrating environmental aspects into every course where appropriate, including assignments addressing sustainability issues during patient counselling; recreating a marketing authorization dossier for a new pharmaceutical product with an environmental risk assessment • Established a teaching event for Green Pharmacy embedded within an existing course; this event included professional lectures on pharmaceuticals in the environment, including emission routes, environmental risk assessment approaches, environmental consciousness in pharmaceutical manufacture, rational drug use and actions to reduce pharmaceutical waste
Approach 2: Climate and health topics embedded within mandatory coursework	
University of California San Francisco School of Pharmacy (USA)^22^	• Developed a 3-hour session focused on climate change, sustainability and pharmaceuticals within a third-year health policy course, including precourse readings and activities, an interactive lecture with a panel of health care professionals and small-group case-based discussions
University of Charleston (USA)^23^	• Hosted 1 lecture, “Pharmacists and the Environment,” as part of a required public health course, including the impacts of extreme weather on health, environmentally friendly interventions for all stages of the medication life cycle, the health care sector’s impact on the United States’ carbon footprint and pharmaceutical drug disposal
Monash University (Australia)^19^	• Launch of a 1-week environmental impact of medicines as part of a fourth-year quality use of medicines course
Approach 3: Climate and health elective courses	
Abo Akademi (Finland)^24^	• Developed a stand-alone annual elective course, “Medicines and the Environment,” that includes lectures, self-study, a 2-hour film and reflection and excursion to a sewage management plant
University of California San Francisco School of Medicine (USA)^25^	• Developed a 10-week student-led elective course, “Earth Health: Sustainability in Health Care,” for health professional students • Aligned course topics with United Nations General Assembly Sustainable Development Goals and covered basic climate change topics, examples of environmental and health consequences of human activities on both global and local scales, legislation and activism to promote actionable change • Included speakers such as clinicians, researchers and public health directors, many with backgrounds in sustainability leadership
Approach 4: Climate and health extracurricular activities	
Planetary Health Report Card (USA)^26^	• Launch of an initiative implemented by medical students to encourage medical schools to incorporate sustainable health care education by measuring each medical school’s “green score” • Piloted at 2 pharmacy schools in the United States to date, with goals to expand
CASCADES Canada – Summer Institute on Sustainable Health Systems (Canada)^27^	• Provide health services and policy trainees (health care professional, public health and health management) with the opportunity to increase awareness of sustainable health systems and current action in Canada • Offers specific training courses
Emerging Leaders for Environmental Sustainability in Healthcare (ELESH) (Canada)^28^	• Collaboration of students from the health faculties and those interested in health and sustainability at the University of Toronto • Aim to raise awareness about issues related to sustainability in health care through events such as case challenges, a trainee committee, a book club and advocacy and research activities

What is needed? First, sustainability as an educational and professional practice requirement is not recognized in Canadian pharmacy professional, educational or accreditation standards. This is a major barrier to establishing environmental sustainability as core course material or as continuing education activities, while also navigating an optimal balance with other program or certification requirements. An environmental sustainability standard will also assist with carving out valuable and coveted space in the curriculum and in professional continuing education standards; it is well known how challenging it can be to incorporate any new program material within pharmacy education due to other emerging priorities.^29^ This calls for collaboration from our educators, regulators and continuing education providers to ensure that environmental sustainability is included as a central pillar of pharmacy standards to raise awareness and enable action.

Second, when environmental sustainability is established as a standard of education and professional practice for emerging and current pharmacists, there can be opportunities for regulators to consider environmental sustainability as a core component of pharmacy accreditation and inspection, while acknowledging that regulators, too, will need education and tools to support the implementation of environmentally sustainable practices in pharmacy. A wide net should be cast to support consultation with pharmacy practitioners, managers, owners, educators and regulators to identify opportunities and barriers for ensuring that environmental sustainability is a mandatory component of pharmacy practice.

Third, existing courses and programs are often led by a proactive “champion,” who may practise within the broader health care landscape surrounding pharmacy practice but not actually be a pharmacist themselves, or who may specifically work at 1 university, organization or institution. While expertise and experience from other professions undoubtedly have their place, this approach to educational development is fragmented, localized and does not encourage wider communities of practice that are pharmacist-led. Significant attention should be given to ensuring pharmacy can develop and support a diverse cadre of its own environmental sustainability leaders within the profession, rather than relying on other health professions to lead it.

As a starting point and with these considerations in mind, we propose launching a national conversation among pharmacy schools in Canada to establish environmental sustainability as a central theme and guiding principle of pharmacy education programs. In the United Kingdom, the ability “to apply the principles, methods and knowledge of . . . sustainable health to medical practice” is now required for all graduating physicians.^30^ Canadian pharmacy schools, regulators and continuing education providers should follow in the same vein when considering that the training of pharmacists will be critical to the sustainability of the health care system at an individual and global level. We suggest the following immediate actions:

1. Bring together pharmacy educators and leaders to develop a consensus among all Canadian pharmacy universities on core environmental sustainability competencies that all pharmacists must possess.
2. Establish requirements for environmental sustainability education and training to be at the core of pharmacy curricula and professional practice standards.
3. Collaborate with national organizations to assist in the development of curriculum, regulatory and continuing education programs focused on environmental sustainability in pharmacy.

Pharmacists are medication stewards who play an integral role as ambassadors of our health care teams and systems. It is critical that our educational programs train pharmacists to practise with environmental sustainability at the core of their work. We write this article with 2 goals: first, to continue to sound the alarm for climate awareness, action and environmentally sustainable practises, and second, to pledge our intention to collaborate with others across Canada on the actions outlined above. The work does not end after writing this short article; it simply is the siren call to take immediate, practical action across a united Canadian pharmacy front for the future training of our pharmacy workforce. In short, the future of our patients, practice and planet depend on it.
